# Mouse Cofactor of BRCA1 (Cobra1) Is Required for Early Embryogenesis

**DOI:** 10.1371/journal.pone.0005034

**Published:** 2009-04-02

**Authors:** Asma Amleh, Sreejith J. Nair, Jianlong Sun, Ann Sutherland, Paul Hasty, Rong Li

**Affiliations:** 1 Department of Molecular Medicine, Institute of Biotechnology, University of Texas, Health Science Center at San Antonio, San Antonio, Texas, United States of America; 2 Department of Cell Biology, University of Virginia School of Medicine, Charlottesville, Virginia, United States of America; The University of Hong Kong, China

## Abstract

**Background:**

Negative elongation factor (NELF) is a four-subunit protein complex conserved from *Drosophila* to humans. *In vitro* biochemical and tissue culture-based studies have demonstrated an important role of NELF in controlling RNA polymerase II (Pol II) pausing in transcription. However, the physiological significance of NELF function is not clear due to the lack of any genetic systems for studying NELF.

**Principal Findings:**

Here we show that disruption of the mouse B subunit of NELF (NELF-B), also known as *cofactor of BRCA1* (*Cobra1*), causes inner cell mass (ICM) deficiency and embryonic lethality at the time of implantation. Consistent with the phenotype of the *Cobra1* knockout (KO) embryos, knockdown of Cobra1 in mouse embryonic stem cells (ESCs) reduces the efficiency of colony formation and increases spontaneous differentiation. *Cobra1*-depleted ESCs maintain normal levels of *Oct4*, *Nanog*, and *Sox2*, master regulators of pluripotency in ESCs. However, knockdown of Cobra1 leads to precocious expression of developmental regulators including *lymphoid enhancer-binding factor 1* (*Lef1*). Chromatin immunoprecipitation (ChIP) indicates that Cobra1 binds to the *Lef1* promoter and modulates the abundance of promoter-bound RNA polymerase.

**Conclusions:**

*Cobra1* is essential for early embryogenesis. Our findings also indicate that *Cobra1* helps maintain the undifferentiated state of mESCs by preventing unscheduled expression of developmental genes.

## Introduction

Inner cell mass (ICM) of the blastocysts is a cluster of cells that gives rise to all the cells of the body. ESCs, which are *in vitro* derivatives of the ICM, maintain the capacity of self-renewal and multi-lineage differentiation. Maintenance of pluripotency or choice of differentiation in both ICM and ESCs is dictated by a transcriptional regulatory circuitry that is composed of a plethora of transcription factors and signal transduction pathways [1], [2]. At the center of the regulatory circuitry are three DNA-binding transcription factors, *Oct4*, *Nanog*, and *Sox2*. These master regulators can coordinately control the expression of two different categories of target genes in ESCs [3], [4]. The first group is activated by the master regulators and is essential for the establishment and maintenance of pluripotency of ESCs. In addition, *Oct4/Nanog/Sox2* repress the expression of a number of developmental genes in order to maintain the undifferentiated state of ESCs. How the master regulators exert the opposing actions on these two types of target genes is not well understood. However, it has been recently shown that most silenced developmental genes are organized in chromatin domains that contain histone modification markers for both transcriptional activation and repression [5]–[7], leading to the notion that the unique chromatin structure helps maintain a silenced yet poised transcriptional state at these loci and renders prompt gene activation in response to developmental cues. Consistent with this notion, Polycomb group (PcG) proteins, which induce condensed chromatin structure, have been implicated in transcriptional repression of developmental genes in ESCs [8], [9].

Cofactor of BRCA1 (COBRA1) was first identified as a BRCA1-interacting protein and subsequently found to be the B subunit of the negative elongation factor complex (NELF-B) [10], [11]. The four-subunit NELF complex was biochemically purified based on its ability *in vitro* to stall RNA polymerase II (RNAPII) in cooperation with the DRB sensitivity-inducing factor (DSIF) at an early stage of transcription elongation [12]. Consistent with *in vitro* findings, tissue culture work indicates that human NELF and its *Drosophila* ortholog can induce transcriptional pausing and attenuate transcription elongation [13], [14]. However, recent whole-genome studies indicate that NELF can also positively regulate a large number of genes in human and flies [15]–[17]. Despite the extensive biochemical and cell culture-based studies, genetic evidence for the physiological importance of COBRA1/NELF is lacking. Using a conditional knockout (KO) mouse model for *Cobra1*, we demonstrate a critical role of *Cobra1* in early embryonic development. Further characterization of *Cobra1* in mouse ESCs indicates that *Cobra1* plays an important role in maintaining the undifferentiated state of ESCs.

## Results

### Whole body deletion of mouse Cobra1 results in embryonic lethality

**Figure 1:**
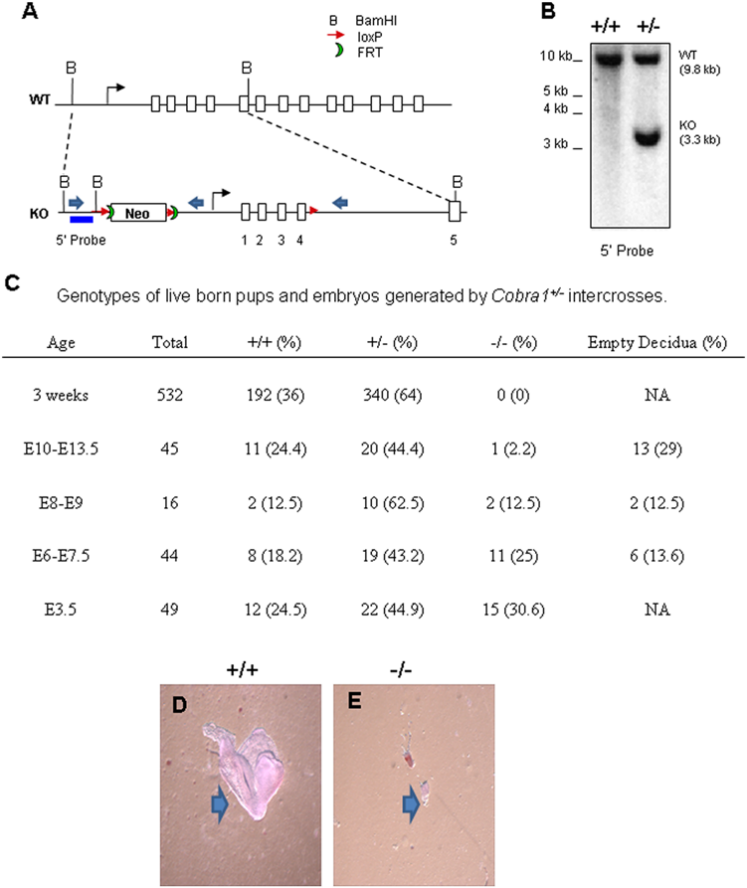
*Cobra1* is essential for early embryonic development. A. Illustration of the wild type *Cobra1* locus (top) and a portion of the targeting construct (bottom). The short (left) and long (right) homology arms encompass genomic regions 1.1 kb upstream of exon 1 and ∼8.4 kb downstream of exon 4, respectively. Also indicated are loxP (red arrows), FRT sites (crescents), exons (open bars), BamH1 sites (B), promoter (solid arrow), 5′ probe (blue bar), and PCR primers (block arrows). B. Southern blot for the BamH1-digested genomic DNA from 3 wk old pups of Cobra1^+/−^ intercross. C. Summary of the genotypes from the *Cobra1^+/−^* intercrosses. D and E. Phenotype of *Cobra1^+/+^* (D) and *Cobra1^−/−^* (E) embryos retrieved at E8 of embryonic development. The block arrows point to embryo proper.

To investigate the *in vivo* function of *Cobra1*, we generated a conditional KO mouse by bracketing the putative promoter and the first four exons of the gene with loxP sites (Fig. 1A). One *floxed-Cobra1* allele in both somatic and germ cells was converted to a null allele by whole-body Cre-mediated recombination (Fig. 1B and Fig. S1A). Heterozygous *Cobra1^+/−^* mice appeared to develop normally. They were fertile and had a normal life span. In multiple adult tissues tested, *Cobra1^+/−^* mice produced approximately half the amount of *Cobra1* mRNA and protein that their wild-type littermates did (Fig. S1B and S1C; data not shown). A similar result was observed with the *Cobra1^+/−^* and *Cobra1^+/+^* embryos (Fig. S1D).

Intercrossing of *Cobra1^+/−^* did not yield any viable progeny that were homozygous for the deletion (3 weeks; Fig. 1C), clearly indicating an essential role of *Cobra1* in embryonic development. To determine the developmental stage at which *Cobra1^−/−^* embryos were lost, embryos from timed mating of *Cobra1^+/−^* were retrieved on various days post-coitum (dpc). Only one out of 45 embryos examined at 10–13.5 dpc was *Cobra1^−/−^*, whereas 2 out of 16 embryos at 8–9 dpc and 11 out of 44 embryos at 6–7.5 dpc carried both deletion alleles (Fig. 1C). Notably, a significant percentage of embryos were reabsorbed, possibly due to homozygous deletion of *Cobra1*. It should be noted that, while we were able to retrieve *Cobra1^−/−^* embryonic materials between 6 and 13.5 dpc, no *Cobra1^−/−^* embryos developed beyond ∼E5 (Fig. 1E). In contrast, wild-type and heterozygous deletion embryos reached the expected developmental age at the time of retrieval (Fig. 1D). Occasionally, we came across *Cobra1^+/−^* embryos that were retarded in development. Taken together, our results clearly demonstrate an essential role for *Cobra1* during early embryonic development.

Human COBRA1 interacts with BRCA1 [10], and the two proteins regulate transcription of a number of genes in concert [15]. Because early embryonic lethality of whole-body *Brca1* KO can be delayed by p53 mutation [18], we sought to determine whether the same were true for *Cobra1^−/−^* embryos. *Cobra1^+/−^; p53^+/−^* compound mice were generated and inter-crossed [19]. No viable *Cobra1^−/−^* mice in the *p53^+/−^* or *p53^−/−^* mutant background were found (Fig. S2). There was no sign of partial rescue by the p53 mutation of *Cobra1^−/−^* embryos beyond E5.5 either (data not shown). Therefore, activation of the p53-mediated checkpoint was an unlikely contributing factor to the lethality associated with the *Cobra1^−/−^* embryos.

### *Cobra1* deletion results in poorly developed inner cell mass

**Figure 2:**
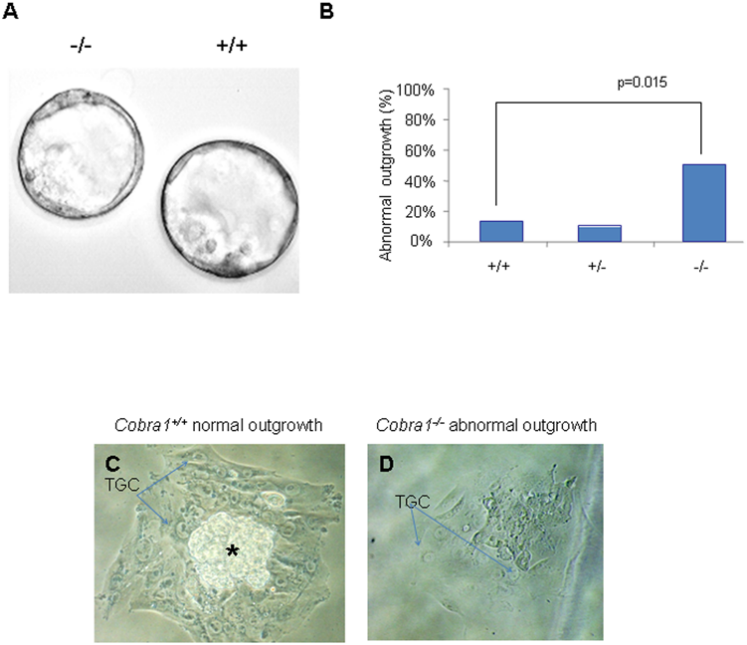
Impaired outgrowth of *Cobra1^−/−^* blastocysts. A. *In vitro* developed blastocysts (*Cobra1^+/+^* and *Cobra1^−/−^*) from two-cell stage embryos. B. *Cobra1* deletion resulted in elevated incidence of outgrowths with defective or no ICM derivatives. C–D. Normal (*Cobra^+/+^*; C) and defective (*Cobra^−/−^*; D) blastocyst outgrowth four days after *in vitro* culture. The ICM derivatives (*) and trophoblast giant cells (TGC) are indicated.

To determine whether *Cobra1^−/−^* embryos at the pre-implantation stage were competent for development, we retrieved embryos at the two-cell stage and cultured them *in vitro* until the blastocyst stage. Approximately 90% of the embryos reached the blastocyst stage while the remaining 10% either stayed at the two-cell stage or resulted in fragmented embryos (data not shown). All three *Cobra1* genotypes were represented in the embryos that developed to the blastocyst stage at the expected Mendelian ratio (E3.5; Fig. 1C). Furthermore, the *Cobra1^−/−^* blastocysts were morphologically indistinguishable from the wild-type counterparts (Fig. 2A). These results indicate that *Cobra1^−/−^* embryos are competent for pre-implantation development and the defect in embryogenesis could lie at a later stage.

The fact that the majority of the embryos fail to proceed to the post-implantation stages could be due to either a defect in hatching from the zona pellucida or a deficiency intrinsic to the ICM in forming the embryo proper. To distinguish these possibilities, we allowed blastocysts from the *Cobra1^+/−^* intercrosses to form outgrowths in culture. The majority of blastocysts (94%) successfully hatched from the zona pellucida (data not shown). After 4 days in culture, all blastocysts produced trophoblast giant cell (TGC) outgrowths but not all contained a discernable ICM. As shown in Fig. 2B and 2C, *Cobra1^−/−^* blastocysts produced a significantly larger number of outgrowths with poorly developed or no ICM than their wild-type and heterozygous counterparts. *Cobra1* deficiency does not appear to affect cell proliferation of the outgrowths, as outgrowths of all three genotypes incorporated the comparable extent of BrdU (Fig. S3). Furthermore, the high rate of abnormal outgrowths associated with *Cobra1^−/−^* blastocysts is unlikely due to delayed growth, because longer periods of *in vitro* culturing (up to 7 days) did not improve the percentage of normal outgrowths from the *Cobra1^−/−^* embryos (data not shown). These findings suggest that *Cobra1* may play an important role in establishment and/or maintenance of the ICM.

### *Cobra1* knockdown impairs the undifferentiated state of mouse embryonic stem cells

**Figure 3:**
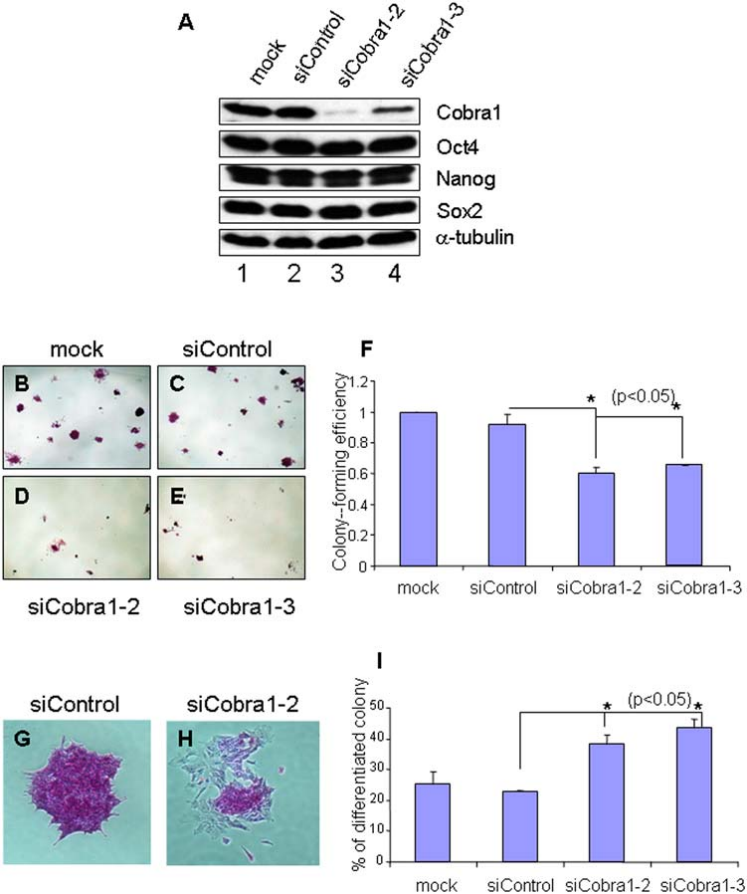
Cobra1 knockdown in mouse ESCs results in reduced colony formation and increased spontaneous differentiation. A. Knockdown of Cobra1 by siRNA does not affect protein levels of Oct4, Nanog, or Sox2. B–E. Cobra1 knockdown reduces the colony formation capability of mESCs. F. Quantitation of the colony formation efficiency in B–E. The value for the mock-transfected cells is set at 1. G–H. Representatives of AP-stained undifferentiated (G) and differentiated (H) ESC colonies. I. Quantitation of the percentage of differentiated colonies.

To better understand the molecular and cellular basis for the function of *Cobra1* in early embryonic development, we examined the role of *Cobra1* in mouse embryonic stem cells (ESCs). As shown in Fig. 3A, siRNA-mediated knockdown significantly depleted ESCs of endogenous Cobra1 protein. Interestingly, levels of all three master regulators (*Oct4*, *Nanog*, and *Sox2*) remained unchanged in the *Cobra1*-knockdown ESCs (Fig. 3A). Compared with the control ESCs (Fig. 3B and 3C), *Cobra1*-depleted ESCs displayed reduced efficiency of colony formation (Fig. 3D–3E; also see quantitation in Fig. 3F). Proliferation rates of the *Cobra1*-knockdown cells were only modestly reduced (Fig. S4), making it an unlikely cause for the reduced efficiency of colony formation. Concomitant with the impaired colony formation, *Cobra1*-knockdown cells tended to form monolayers of loosely associated cells with a fibroblastic morphology (compare Fig. 3G and 3H). Furthermore, Cobra1-knockdown cells displayed diminished staining for alkaline phosphatase (AP), an established marker for ESC (Fig. 3G–3I). These findings suggest that *Cobra1* helps maintain the undifferentiated state of mESCs.

### *Cobra1* depletion increases the expression of development-associated genes

Given the well-documented transcriptional regulatory activity of NELF, we conducted a microarray experiment using ESCs that were transiently transfected with either control or *Cobra1*-specific siRNA oligos. Using a fold change of 1.5 (log_2_) and p value of 0.05 as the cutoff, we identified a total of 334 and 403 up- and down-regulated genes, respectively, in the Cobra1-knockdown cells (Table S1). Gene ontology (GO) analysis indicates that developmental genes are over-represented among the up-regulated genes in Cobra1-knockdown cells (Fig. S5). Interestingly, a significant number of the developmental genes have been previously shown to be occupied by at least one of the three master pluripotency regulators [3] (Table S2).

**Figure 4:**
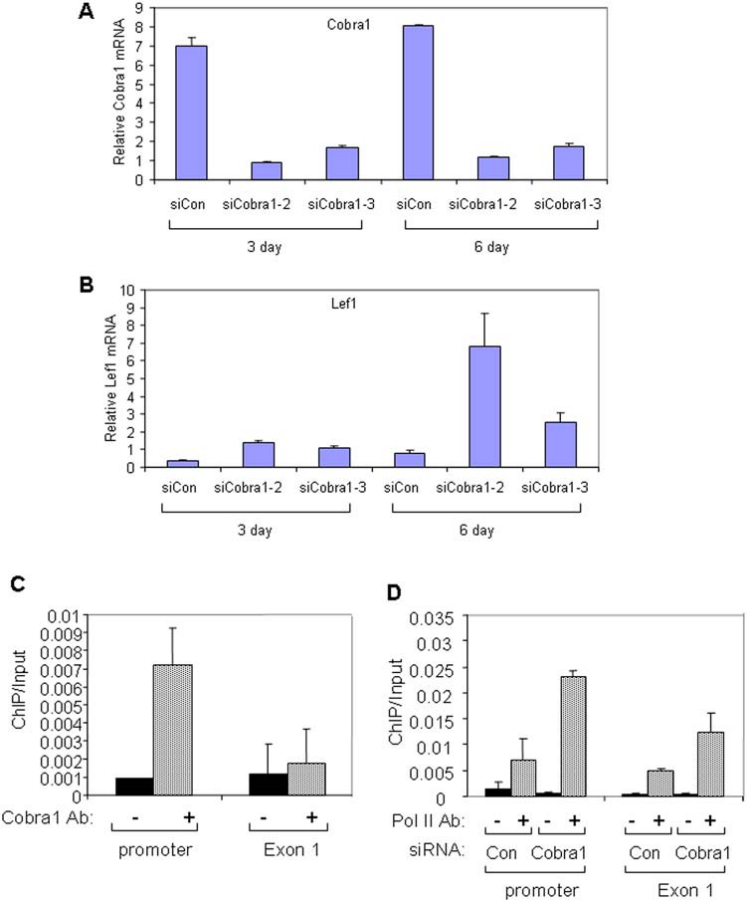
Cobra1 is required for transcriptional repression of *Lef1* in ESCs. A. Real-time RT-PCR of *Cobra1* mRNA in control and *Cobra1*-knockdown cells 3 and 6 days after siRNA transfection. B. *Lef1* mRNA in control and *Cobra1*-knockdown cells. C. Cobra1 ChIP at the promoter and exon 1 of the *Lef1* gene in parental ESCs. Preimmune antiserum was used as the negative control. D. Pol II ChIP at the promoter and exon 1 of the *Lef1* locus in control and *Cobra1*-knockdown ESCs six days after transfection.

Lymphoid enhancer-binding factor 1 (*Lef1*), a key transcription factor in the Wnt-mediated signal transduction pathway [20], was identified by the microarray study as the most significantly up-regulated developmental genes in the *Cobra1*-knockdown cells. We verified the effect of *Cobra1* knockdown on *Lef1* mRNA by quantitative RT-PCR using two independent *Cobra1* siRNA oligos (Fig. 4A and 4B). Furthermore, chromatin immunoprecipitation (ChIP) demonstrates a physical association of Cobra1 with the *Lef1* promoter region (Fig. 4C), suggesting a direct impact of Cobra1 on *Lef1* transcription. To ascertain the involvement of *Cobra1* in modulation of *Lef1* mRNA synthesis, Pol II ChIP was conducted in control and Cobra1-knockdown ESCs. As shown in Fig. 4D, the amount of Pol II at both the promoter and first exon of the *Lef1* gene was substantially elevated in *Cobra1*-knockdown cells, suggesting that Cobra1 modulates the presence of Pol II at the promoter-proximal region of the *Lef1* gene.

## Discussion

Recent studies of genome-wide transcription suggest that Pol II pausing is a highly conserved and widespread phenomenon in eukaryotes [21]–[24]. Among the limited number of Pol II-pausing factors identified so far, NELF is unique in that it is only present in higher eukaryotes[12], [25]. Studies of NELF have been predominantly focused on its biochemical and molecular function in Pol II pausing and transcriptional regulation. What is conspicuously lacking is any genetic evidence for the physiological outcomes of NELF-mediated polymerase pausing and gene regulation. To fill this gap in the knowledge of NELF, we generated a conditional mouse KO model for *Cobra1/NELF-B*. We demonstrate an essential role of *Cobra1* in early embryogenesis. Given that human COBRA1 is known to function as an integral component of the NELF complex and that levels of individual NELF subunits are interdependent [11]–[13], [17], [25], [26], it is highly likely that the entire mouse NELF complex is critical for embryonic development.

Our study suggests that *Cobra1* facilitates the maintenance of the undifferentiated state of mESCs. One possible underlying mechanism is the *Cobra1*-mediated repression of development-associated genes. It remains to be determined how many of the developmental genes identified by the microarray study are direct targets of NELF and which of these potential target genes critically mediates the NELF function in ESCs. However, our data indicate that Cobra1 is physically associated with at least the promoter region of the *Lef1* gene. Lef1 forms heterodimers with its DNA-binding partners Tcf proteins; and the Lef1/Tcf-mediated Wnt/β-catenin signaling is pivotal to the functions of multipotent stem cells in the intestine, skin, and the immune system [27]. Furthermore, Tcf3 co-occupies a large number of promoters with the master regulators Oct4 and Nanog in mESCs [28]; and depletion of Tcf3 causes increased expression of master regulators and delayed differentiation [28], [29]. In addition, *Lef1* has been implicated in trophoblast lineage differentiation of mESCs [30]. Thus, elevated expression of *Lef1* in *Cobra1*-knockdown ESCs could contribute to the observed spontaneous differentiation in ESCs, impaired outgrowth, and early embryonic lethality.

The overt phenotype associated with *Cobra1* KO/knockdown is reminiscent of those associated with disruption of the master regulator genes [31]. However, unlike the master regulators, *Cobra1* expression is not limited to pluripotent stem cells, suggesting that its function is necessary but not sufficient for pluripotency. Within the context of ESCs, an important function of *Cobra1* may be to help maintain developmental genes in a repressed yet poised transcriptional state. Consistent with this notion, *Cobra1* depletion leads to elevated transcription of multiple developmental genes in ESCs without affecting the levels of *Oct4*, *Nanog*, or *Sox2*. Therefore, *Cobra1* most likely exerts its action in conjunction with, or independent of the master regulators. As *Cobra1* is not known to bind DNA by itself, it may be recruited to its target genes by the three master regulators. Alternatively, *Cobra1* could repress transcription through its putative interactions with other DNA-binding transcription repressors that play critical roles in ESC functions [32].

Recent genome-wide analyses uncovered an unexpected transcriptional and chromatin status of the developmental genes that are repressed by the master regulators in ESCs [7]. The majority of these genes experience transcription initiation, as evidenced by the presence of histone modification marks that are associated with active transcription initiation. Furthermore, Pol II can be detected at the promoter-proximal region of these transcriptionally inactive genes. The well-established biochemical function of NELF in polymerase pausing during transcription elongation would be consistent with a role of Cobra1 in keeping developmental genes in a poised transcriptional state. In this regard, it is somewhat surprising that *Cobra1* knockdown significantly increases the total amount of promoter-associated polymerase at the *Lef1* promoter-proximal region. Although it remains to be seen whether Cobra1 could regulate other putative targets in a similar fashion, our finding raises an intriguing possibility that the function of NELF may not be limited to modulation of transcription elongation.

It has been shown that NELF represses transcription of human JunB by reducing the overall polymerase density at the promoter region [33]. Recent data also show that *Drosophila* NELF can activate transcription by preventing nucleosomal assembly in the vicinity of the transcription initiation site [16]. It is worth noting that approximately half of the genes in our microarray study were down-regulated by *Cobra1* knockdown. Further investigation of Cobra1-mediated transcription regulation in ESCs will provide a more comprehensive picture of the underlying mechanism(s) by which *Cobra1* contributes to the maintenance of the undifferentiated state of ESCs.

## Materials and Methods

### Ethics Statement

All animal procedures were approved by the Institutional Animal Care and Use Committee.

### Generation of the floxed and deleted *Cobra1* alleles

Mice described are of mixed genetic background (C57BL/6×129/SvJ) unless specified. A 1.1 kb DNA fragment 5′ to exon 1 of *Cobra1* and 8.4 kb fragment 3′ to exon 4 were sub-cloned into a targeting vector. A loxP site was inserted 3′ to exon 4 and a *loxP*/*FRT*-flanked neomycin (neo) resistance cassette was inserted 5′ to exon 1 (Fig. 1A). The targeting construct was linearized with NotI and electroporated into iTL1 129Svev ES cells (inGenious Targeting Laboratory). DNA from antibiotic-resistant clones was digested with BamHI and subjected to Southern blot analysis. Genomic integration of the *loxP*-containing cassette was confirmed by PCR amplification (PCR primers A3 and N1; Table S3) and by sequencing (primer WW3). Positive clones were microinjected into C57Bl/6 blastocysts and transferred into CD-1 foster mothers. The resulting male chimeras were mated with wild-type C57Bl/6 females to test for germline transmission. F1 agouti mice were genotyped by PCR. The F1 agouti mice of *Cobra1^fl-neo/+^* genotype were crossed with *Flp-deleter* transgenic mice (Stk#003946; Jackson Laboratory) to remove the Neo cassette by the FLP-mediated recombination [34]. The single floxed *Cobra1* allele was converted to a null allele by Cre-mediated recombination (EIIa-Cre) [35].

### Embryo Recovery

*Cobra1^+/−^* females were super-ovulated by intra-peritoneal injections of 5 IU of pregnant mare's serum gonadotrophin (PMSG, National Hormone & Peptide Program, California) and 46–48 hours later, human chorionic gonadotrophin (hCG, MP Biomedicals, Inc.). Super-ovulated females were bred with *Cobra1^+/−^* males. Oviducts were isolated from female mice 40–48 hours after super-ovulation and flushed with FHM medium (Chemicon). Embryos were washed three times in FHM medium, twice in KSOM +AA with D-glucose (Chemicon), and incubated in KSOM droplets at 37°C under 5% CO_2_. Droplets of KSOM were covered with embryo culture-tested mineral oil (Sigma). Blastocysts were removed from KSOM, washed with blastocyst outgrowth media [36], and seeded separately into 24-well plates. The blastocysts outgrowths were scored 4–5 days later, using an inverted microscope.

### Genotyping

Genotypes of mice adults and embryos were identified using genomic DNA isolated from mouse-tails and whole pre-implantation embryos, respectively. DNA from tail snips was obtained by the salting-out [37] or NaOH procedure [38]. Purified genomic DNA was digested with BamHI and subjected to Southern blot analysis, using a 1 kb probe that corresponds to a genomic region upstream of exon 1 of the *Cobra1* gene (Fig. 1A). To distinguish between the wild type and KO *Cobra1* alleles by PCR, we used a common upstream primer (CobP; Table S3) in combination with either a downstream primer specific for the wild type allele (CobWr) or the KO allele (CobNr). Due to the scarcity of the material retrieved from pre-implantation and blastocyst outgrowths, real-time PCR was used to determine the genotype of the developing embryos.

### ES cell culture

Undifferentiated AB2.2 ES cells were maintained in high glucose Dulbecco's Modified Eagle's Medium (DMEM, Gibco) supplemented with 15% fetal bovine serum (Gibco), 2 mM L-glutamine (Gibco), 0.1 mM 2-mercaptoethanol (Sigma-Aldrich), 50 U/ml penicillin, 50 µg/ml streptomycin (Pen-strep, Gibco), and 1000 U/ml ESGRO-LIF (Millipore). The cells were grown on 0.1% gelatin-coated dishes.

### Antibodies

The following commercially available antibodies were used in this study; Oct4 (Abcam, ab19857), Sox-2 (Santa Cruz biotechnology, sc-17320), Nanog (Bethyl laboratories, A300-397A), tubulin (Calbiochem, CP06), RNA Pol II (Abcam, ab5408). Anti-Cobra1 rabbit polyclonal antibody was generated by immunizing rabbits (Covance) with purified His-tagged Cobra1 protein. The COBRA1 monoclonal antibody used in immunoblotting has been described previously [13].

### siRNA knockdown

Transfections with siGenome duplexes against *Cobra1* (Dharmacon) were performed in suspension using Lipofectamine 2000 (Invitrogen) according to manufacturer's instructions. The cells were plated in 6 well plates at a density of 4.5×10^5^ cells per well. RNA was harvested for analysis 3 days after the first transfection. For the 6-day time point, cells were re-transfected with the same siRNA oligos 72 hrs after the first transfection. In all knockdown experiments siGenome non-targeting siRNA (D-001210-0X, Dharmacon) and 1× siRNA dilution buffer (Dharmacon) were used as negative controls.

### RNA extraction, cDNA synthesis, and real-time PCR

RNA was extracted with Trizol reagent (Invitrogen). cDNA was synthesized with 1 µg of total RNA from ESCs using the ImPromII Reverse Transcription System (Promega) and random primers. For RNA from mouse tissues, Superscript II reverse transcriptase (Invitrogen) kit was used for cDNA synthesis. cDNA from pre-implantation embryos was obtained by using the Cells-to-cDNA II kit (Ambion) according to manufacturer's protocol. Quantitative PCR was conducted using an ABI Prism 7900 machine. Expression levels were normalized against either *Gapdh* (mouse tissue) or 18 s ribosomal RNA (ESCs). Results were confirmed with at least three independent experiments.

### Immunoblotting

After extracting RNA from embryonic tissue samples using Trizol (Invitrogen), the organic phase was processed for DNA extraction and subsequently protein extraction. Alternatively, ESCs or frozen tissue samples were lysed and homogenized in Laemmli buffer (50 mM Tris pH 6.8, 2% SDS, 10% glycerol, 100 mM DTT) that contains a cocktail of protease inhibitors. Protein content was measured using the BCA Protein Assay Kit (Pierce). Immunoblotting was conducted using chemiluminescence (SuperSignal West Pico, Pierce) according to the manufacturer's instructions.

### Microarray experiment, statistical analysis, and gene ontology analysis

AB2.2 cells in duplicate were transfected with control or *Cobra1* siRNA. Microarray was conducted by Nimblegen using a mouse 4-plex expression array (MM8 60mer expr ×4). The data were analyzed using Genespring 9 software (Agilent Technologies). Gene ontology classification was conducted using David bioinformatics resources (http://david.abcc.ncifcrf.gov/). Functional significance of each gene clusters were determined based on the enrichment score.

### Chromatin Immunoprecipitation (ChIP)

ESCs were cross-linked with 1% formaldehyde for 10 min, treated with glycine at a final concentration of 0.125 M for 5 min at room temperature, and lysed in lysis buffer (5 mM HEPES; pH 9.0; 85 mM KCl, 0.5% Triton X-100) for 15 min on ice. Nuclei were resuspended in nuclei lysis buffer (50 mM Tris-HCl; pH 8.0, 10 mM EDTA; pH 8.0, 1% SDS), and the cross-linked DNA was sonicated for 10 min (with 30 s on/off cycles) using Bioruptor (Diagenode) according to manufacturers instruction. The supernatant was used for ChIP as previously described [39].

### Colony formation and alkaline phosphatase (AP) staining

ESCs were plated in triplicate on gelatin-coated 6-well plates and allowed to grow for 5 days. The colonies were stained using the StemTAG alkaline phosphatase staining kit (CBA-300; Cell Biolabs) according to manufacturer's instructions. The colonies on 10 randomly chosen fields at ×4 magnification were counted per well and classified into undifferentiated or differentiated groups based on the morphology. The colony number for mock-transfected cells was set at 100%. The value in the figure is mean +/− standard deviation. The data were subjected to student's t-test using Sigma Plot 8.0.

## Supporting Information

Figure S1 A. PCR-based genotyping of 3 week-old mice from Cobra1+/− intercrosses. DNA samples were subjected to PCR analysis using CobP, CobWr, CobNr (see Table S3). The wild type (wt) and knockout alleles generate 350 bp and 550 bp PCR fragments, respectively. B–C. Quantitative RT-PCR analysis of Cobra1 mRNA levels in the testicular tissue (B), ovaries (C) harvested from wt and Cobra1+/− mice. D. Cobra1 immunoblot of lysates from wt and Cobra1+/− embryos at E8.5.
(1.50 MB TIF)

Figure S2 The embryonic lethality of Cobra1 knockout mice cannot be rescued by p53 mutations.
(0.92 MB TIF)

Figure S3 Cobra1-deficient outgrowths display comparable proliferation rates as the controls. Blastocysts that had been grown in culture for three days were incubated with BrdU at 10 mM (B9285-1G; Sigma) for 12–16 hours. Outgrowths were fixed with 4% paraformaldehyde in phosphate buffered saline (PBS). Following cell permeablization with 0.4% Triton X-100 for 5 min at room temperature (RT), DNA was denatured by incubation in 2 N HCl for 1 hour in the dark at 37°C and subsequently neutralized with 0.1 M sodium borate (pH 8.5). The outgrowths were blocked with 10% BSA/PBS for 10 minutes. BrdU incorporation was detected by incubation with a mouse monoclonal anti-BrdU antibody (1∶50; Alexa Fluor 594, Roche) in blocking solution for 1 hour in the dark at 37°C. Shown are representatives of a total of 29 outgrowths analyzed.
(2.33 MB TIF)

Figure S4 Growth curves for mock-, control siRNA, and two Cobra1 siRNA-transfected ESCs. For measuring cell proliferation, cells were plated in triplicate at a density of 2000 cells/well in a 96-well plate. Cell proliferation was measured from day 1 to 5 using CellTiter96 Aqueous One Solution Cell proliferation assay (Promega) according to manufacturer's instructions.
(1.08 MB TIF)

Figure S5 Gene Ontology of the microarray result from up-regulated genes in Cobra1 knockdown ESCs.
(1.33 MB TIF)

Table S1 Genes in ES cells that are up- or down-regulated by COBRA1 knockdown
(0.32 MB XLS)

Table S2 Developmental genes that are up-regulated in Cobra1 knockdown ESCs. Also shown are association of the promoter region of each gene with the three master regulators as shown by a previously published study (Boyer, L.A. et al. Cell 122: 947–956).
(1.34 MB TIF)

Table S3 Primers used in the study
(1.33 MB TIF)
