# Supplementary figures and images for: Mouse Cofactor of BRCA1 (Cobra1) Is Required for Early Embryogenesis
Source: PLoS One. 2009 Apr 2;4(4):e5034. doi: 10.1371/journal.pone.0005034 (PMC2661135; doi:10.1371/journal.pone.0005034)

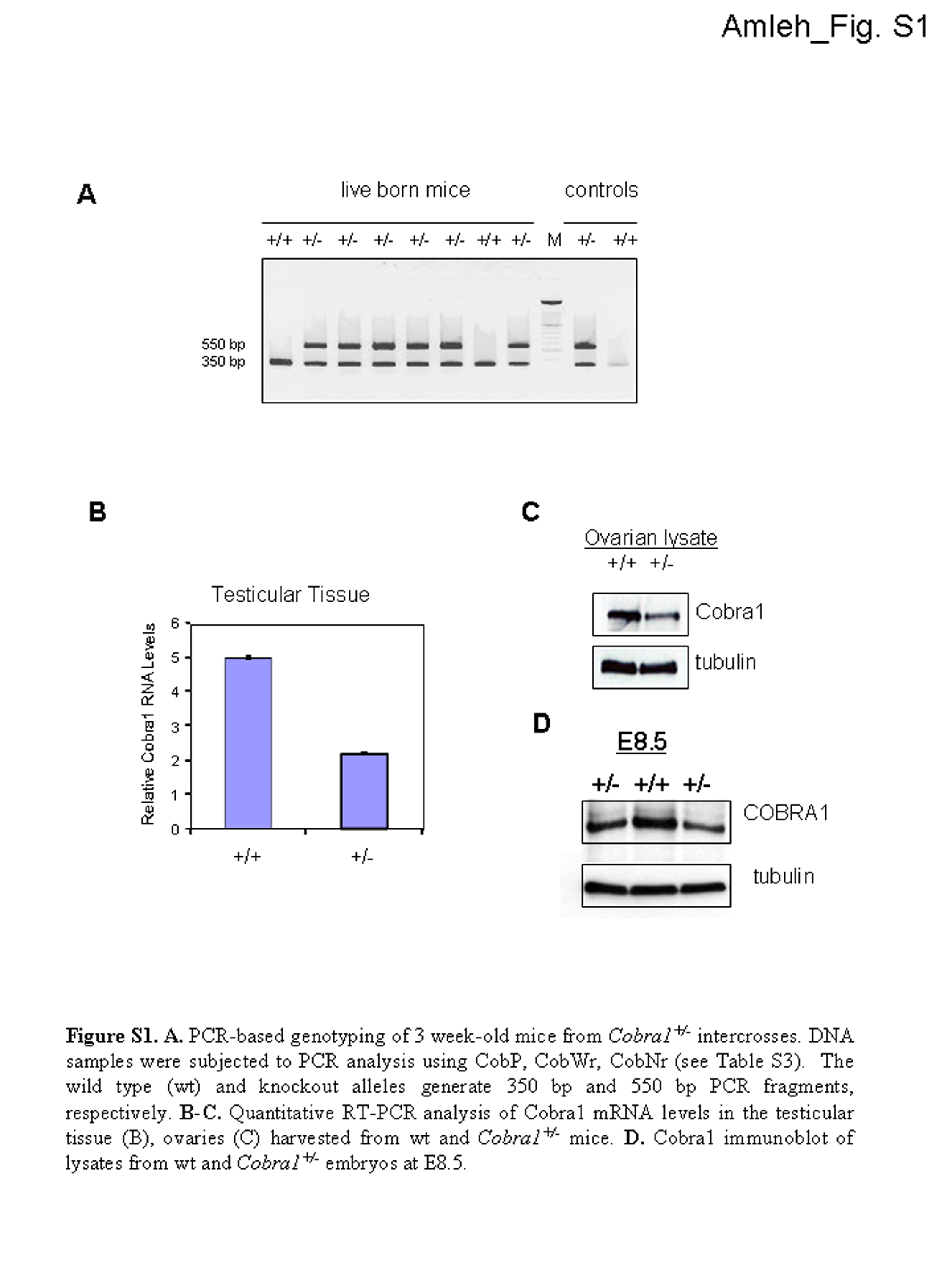

Supplement: Figure S1 — A. PCR-based genotyping of 3 week-old mice from Cobra1+/− intercrosses. DNA samples were subjected to PCR analysis using CobP, CobWr, CobNr (see Table S3). The wild type (wt) and knockout alleles generate 350 bp and 550 bp PCR fragments, respectively. B–C. Quantitative RT-PCR analysis of Cobra1 mRNA levels in the testicular tissue (B), ovaries (C) harvested from wt and Cobra1+/− mice. D. Cobra1 immunoblot of lysates from wt and Cobra1+/− embryos at E8.5. (1.50 MB TIF) [file pone.0005034.s001.tif]

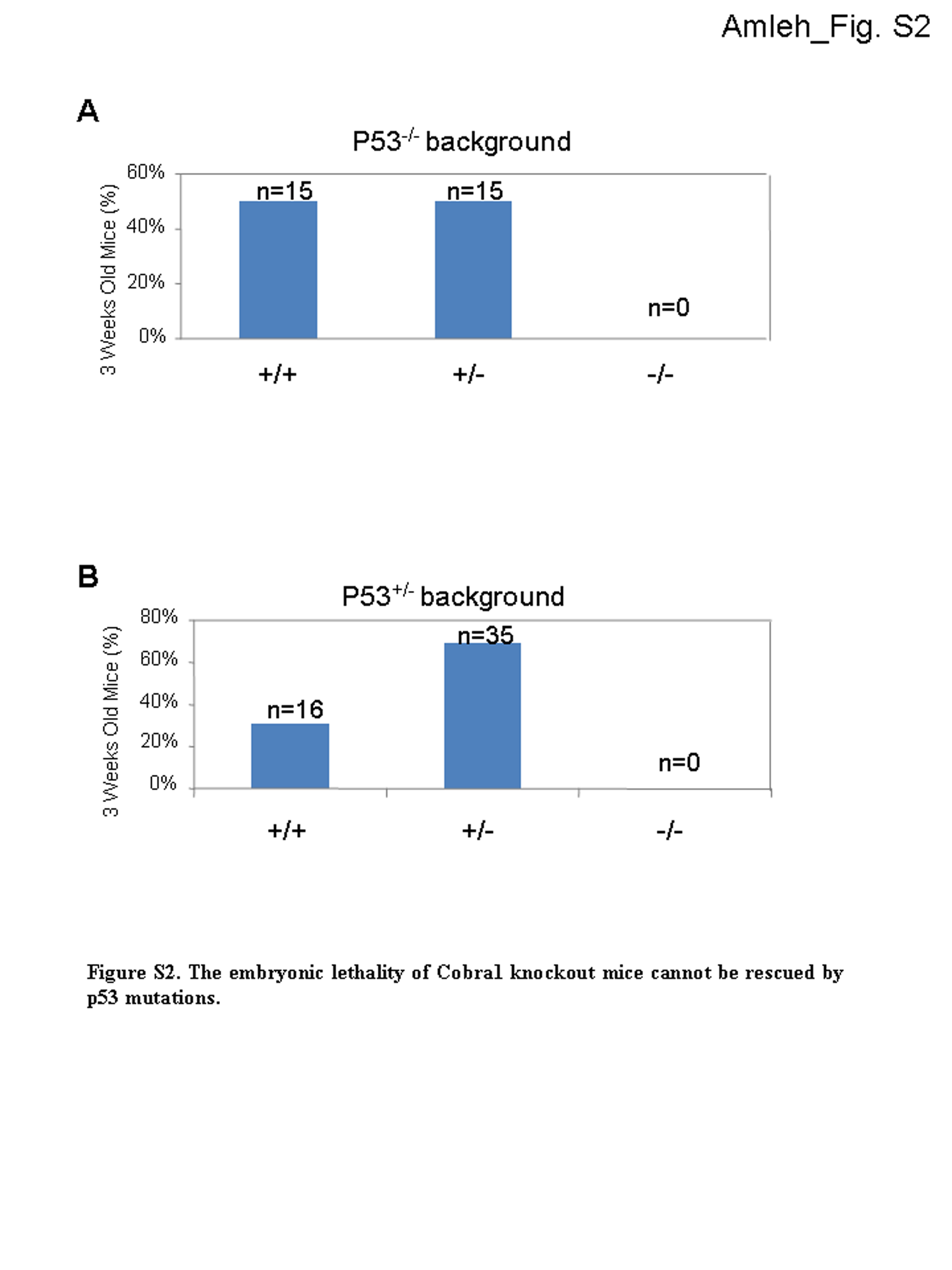

Supplement: Figure S2 — The embryonic lethality of Cobra1 knockout mice cannot be rescued by p53 mutations. (0.92 MB TIF) [file pone.0005034.s002.tif]

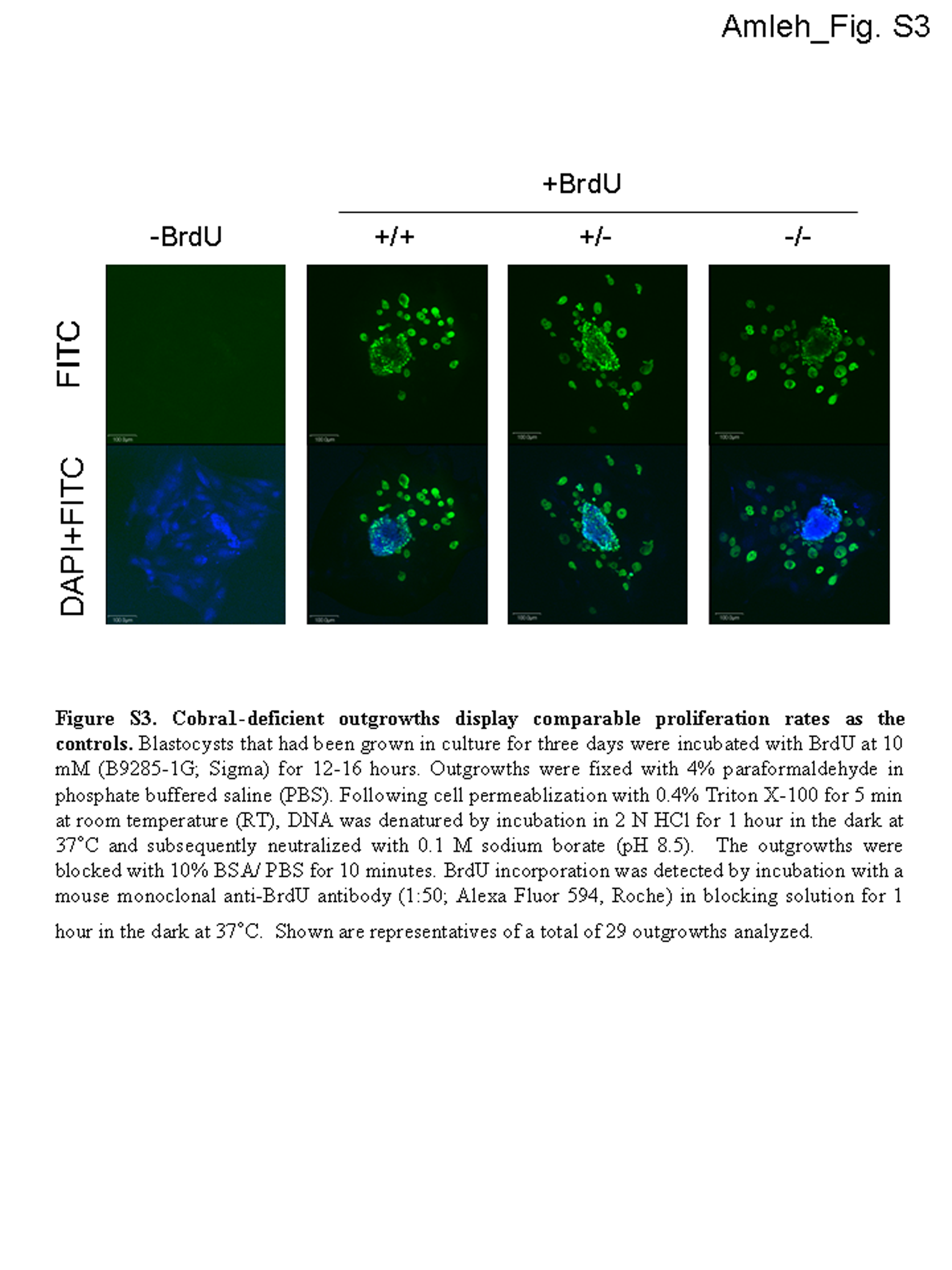

Supplement: Figure S3 — Cobra1-deficient outgrowths display comparable proliferation rates as the controls. Blastocysts that had been grown in culture for three days were incubated with BrdU at 10 mM (B9285-1G; Sigma) for 12–16 hours. Outgrowths were fixed with 4% paraformaldehyde in phosphate buffered saline (PBS). Following cell permeablization with 0.4% Triton X-100 for 5 min at room temperature (RT), DNA was denatured by incubation in 2 N HCl for 1 hour in the dark at 37°C and subsequently neutralized with 0.1 M sodium borate (pH 8.5). The outgrowths were blocked with 10% BSA/PBS for 10 minutes. BrdU incorporation was detected by incubation with a mouse monoclonal anti-BrdU antibody (1∶50; Alexa Fluor 594, Roche) in blocking solution for 1 hour in the dark at 37°C. Shown are representatives of a total of 29 outgrowths analyzed. (2.33 MB TIF) [file pone.0005034.s003.tif]

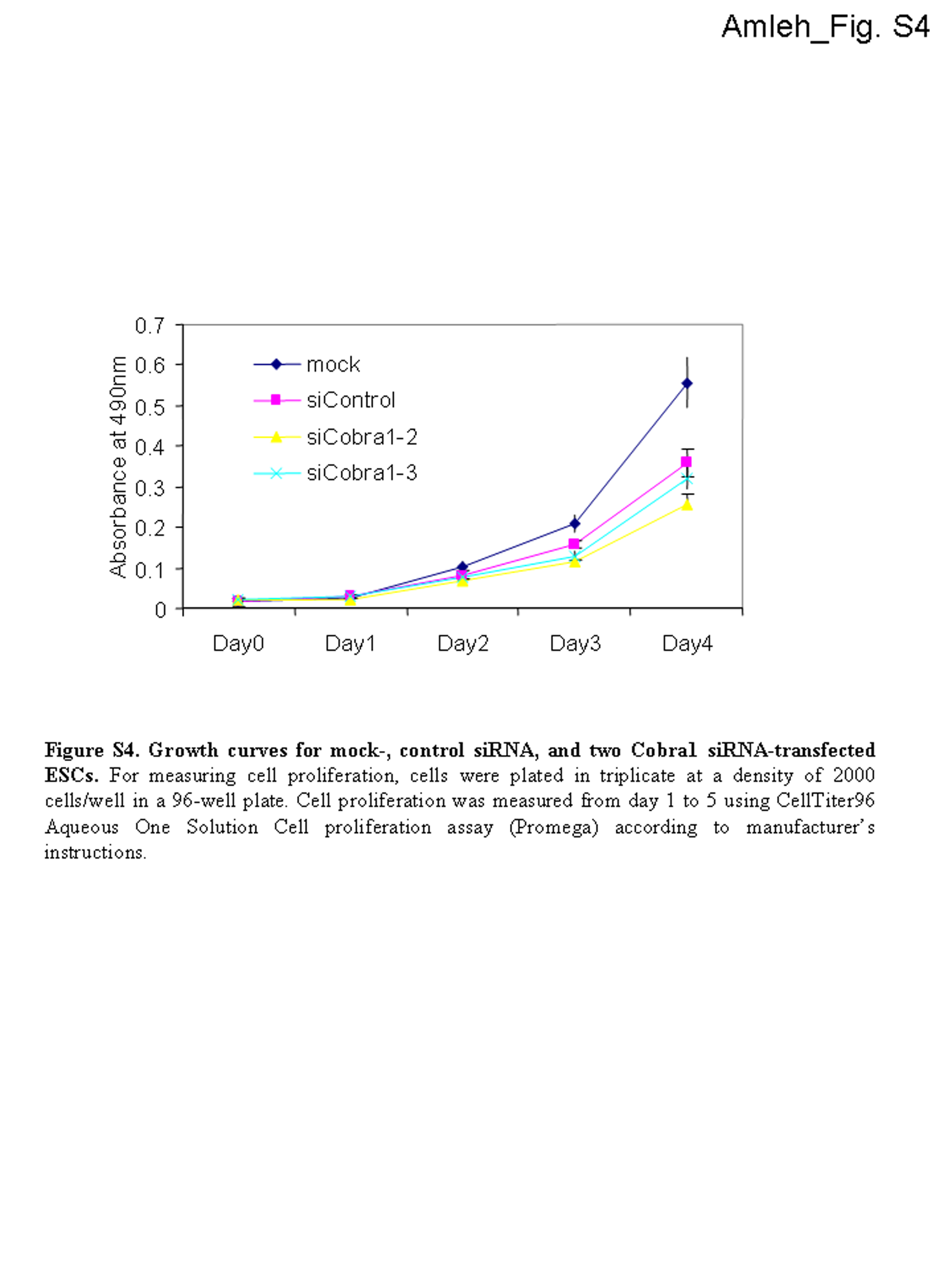

Supplement: Figure S4 — Growth curves for mock-, control siRNA, and two Cobra1 siRNA-transfected ESCs. For measuring cell proliferation, cells were plated in triplicate at a density of 2000 cells/well in a 96-well plate. Cell proliferation was measured from day 1 to 5 using CellTiter96 Aqueous One Solution Cell proliferation assay (Promega) according to manufacturer's instructions. (1.08 MB TIF) [file pone.0005034.s004.tif]

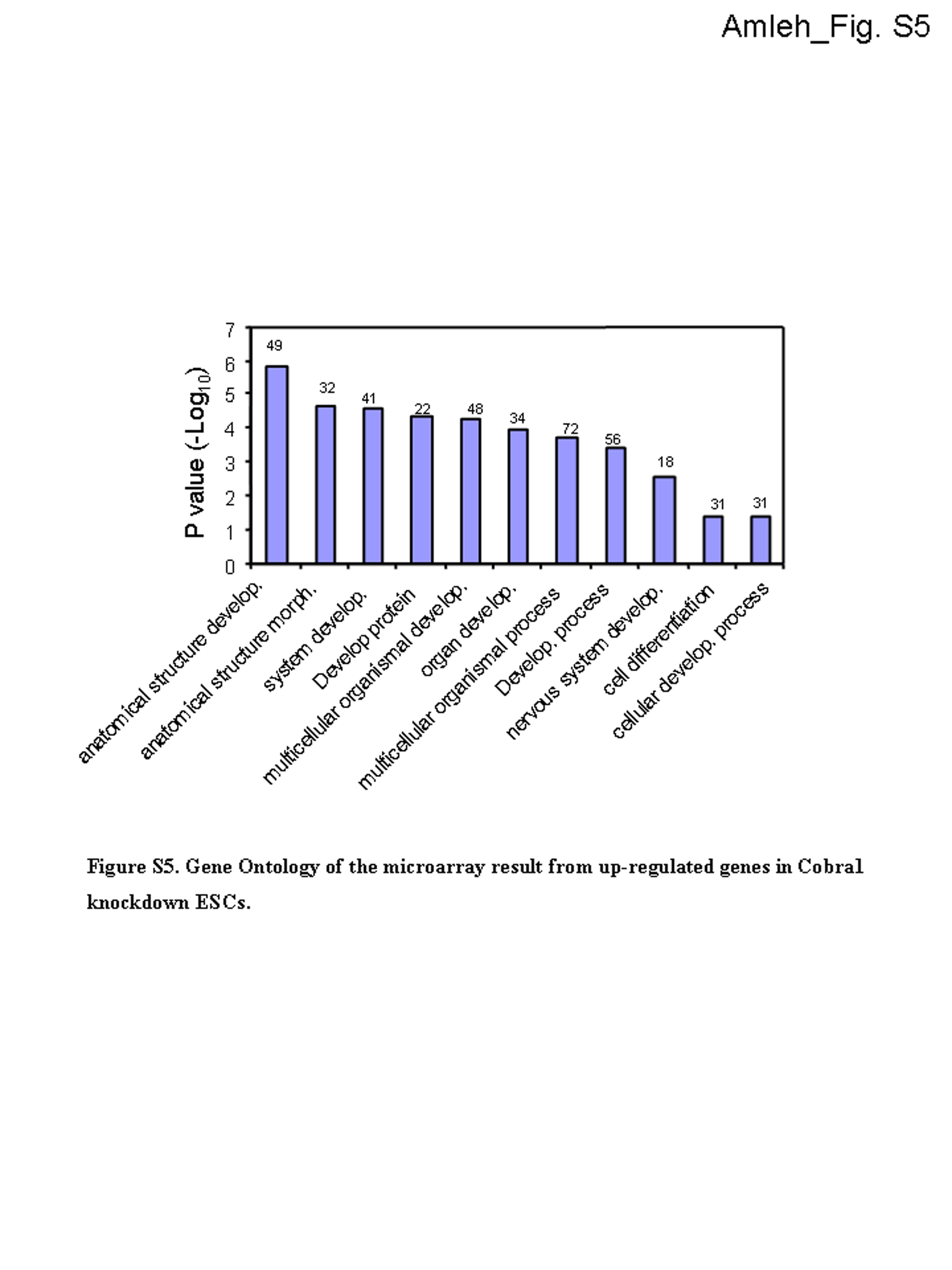

Supplement: Figure S5 — Gene Ontology of the microarray result from up-regulated genes in Cobra1 knockdown ESCs. (1.33 MB TIF) [file pone.0005034.s005.tif]

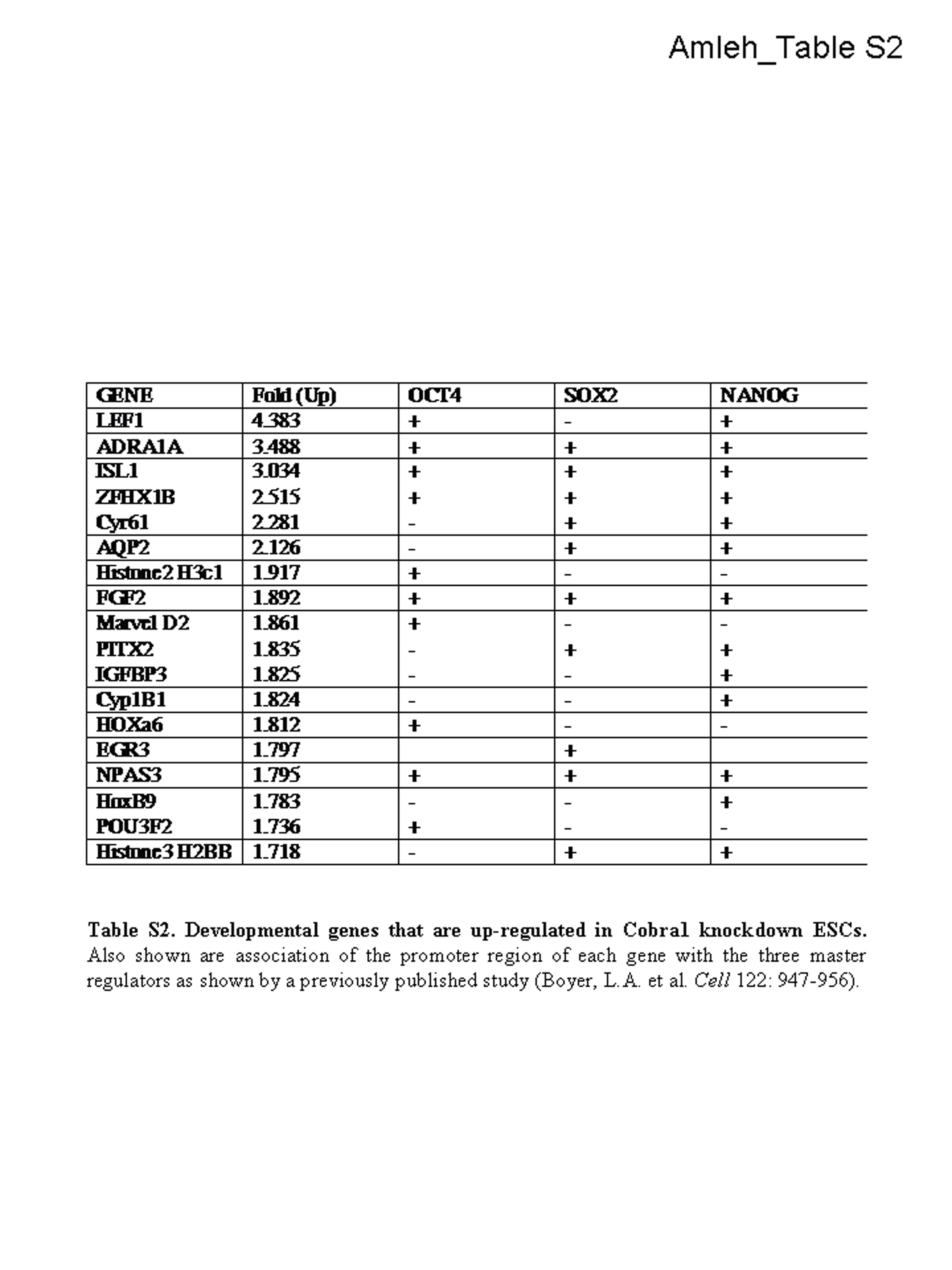

Supplement: Table S2 — Developmental genes that are up-regulated in Cobra1 knockdown ESCs. Also shown are association of the promoter region of each gene with the three master regulators as shown by a previously published study (Boyer, L.A. et al. Cell 122: 947–956). (1.34 MB TIF) [file pone.0005034.s007.tif]

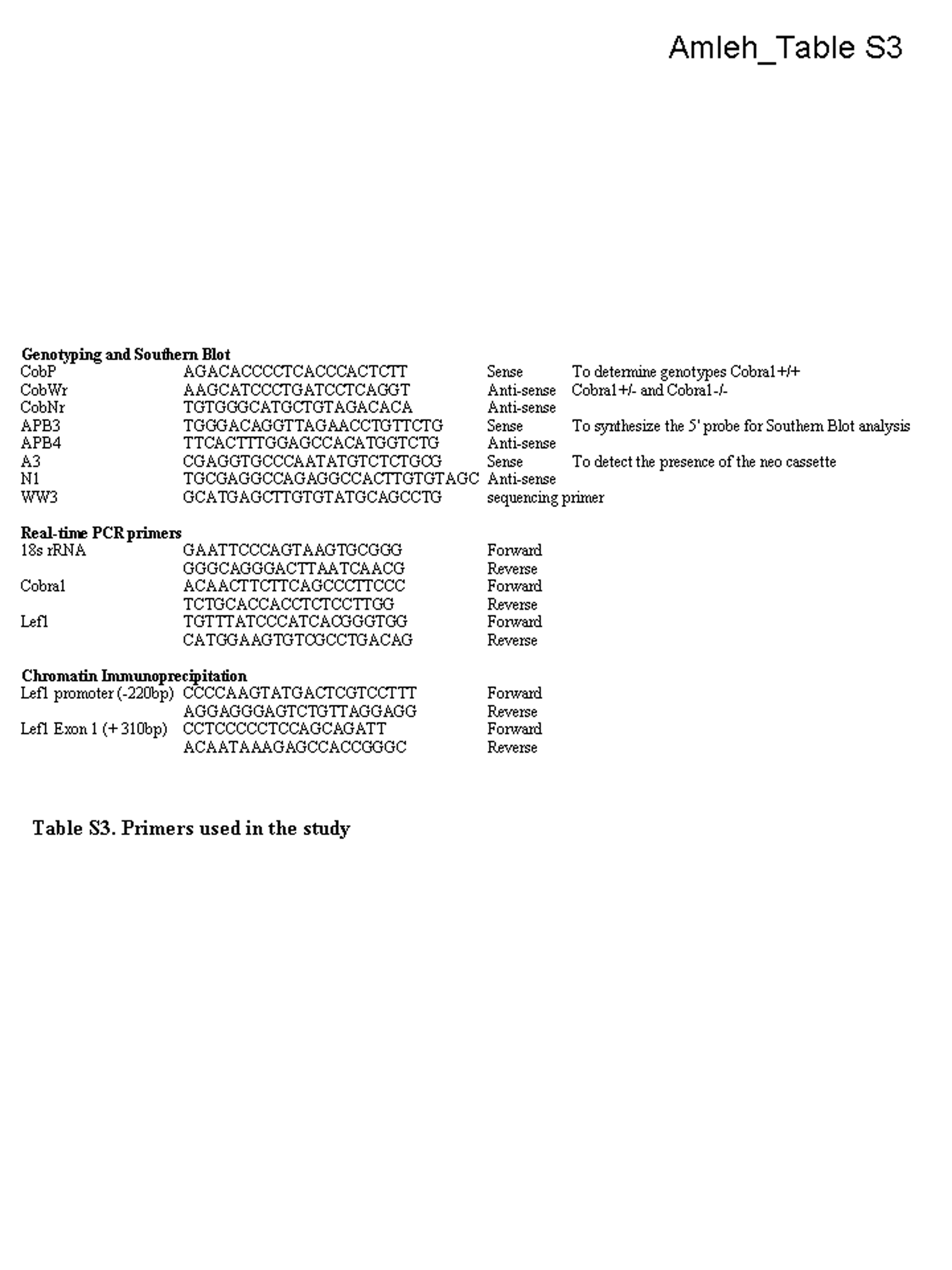

Supplement: Table S3 — Primers used in the study (1.33 MB TIF) [file pone.0005034.s008.tif]
